# Multifunctional Gas and pH Fluorescent Sensors Based on Cellulose Acetate Electrospun Fibers Decorated with Rhodamine B-Functionalised Core-Shell Ferrous Nanoparticles

**DOI:** 10.1038/s41598-019-57291-0

**Published:** 2020-01-15

**Authors:** Afroditi Petropoulou, Slavko Kralj, Xenofon Karagiorgis, Ioanna Savva, Emilios Loizides, Myrofora Panagi, Theodora Krasia-Christoforou, Christos Riziotis

**Affiliations:** 1 National Hellenic Research Foundation, Theoretical and Physical Chemistry Institute, Photonics for Nanoapplications Laboratory, Athens, 11635 Greece; 2 University of Peloponnese, Department of Informatics and Telecommunications, Tripolis, 22100 Greece; 3 Department for Materials Synthesis, Jožef Stefan Institute, Jamova 39, 1000 Ljubljana, Slovenia; 4 Nanos SCI (Nanos Scientificae Ltd), Teslova 30, 1000 Ljubljana, Slovenia; 5 University of Cyprus, Department of Mechanical and Manufacturing Engineering, Nicosia, 1678 Cyprus

**Keywords:** Sensors and biosensors, Organic-inorganic nanostructures

## Abstract

Ferrous core-shell nanoparticles consisting of a magnetic γ-Fe_2_O_3_ multi-nanoparticle core and an outer silica shell have been synthesized and covalently functionalized with Rhodamine B (RhB) fluorescent molecules (γ-Fe_2_O_3_/SiO_2_/RhB NPs). The resulting γ-Fe_2_O_3_/SiO_2_/RhB NPs were integrated with a renewable and naturally-abundant cellulose derivative (i.e. cellulose acetate, CA) that was processed in the form of electrospun fibers to yield multifunctional fluorescent fibrous nanocomposites. The encapsulation of the nanoparticles within the fibers and the covalent anchoring of the RhB fluorophore onto the nanoparticle surfaces prevented the fluorophore’s leakage from the fibrous mat, enabling thus stable fluorescence-based operation of the developed materials. These materials were further evaluated as dual fluorescent sensors (i.e. ammonia gas and pH sensors), demonstrating consistent response for very high ammonia concentrations (up to 12000 ppm) and fast and linear response in both alkaline and acidic environments. The superparamagnetic nature of embedded nanoparticles provides means of electrospun fibers morphology control by magnetic field-assisted processes and additional means of electromagnetic-based manipulation making possible their use in a wide range of sensing applications.

## Introduction

Nanoparticle-based systems containing more than one functional components represent an active research field having a great potential in numerous technological applications^1^. Among others, magnetic core-shell nanoparticles offer new opportunities in the biomedical field, catalysis and sensing^2–10^. In particular, fluorescent-functionalized silica-coated core-shell magnetic nanoparticles attract high attention in imaging and sensing applications. In such multifunctional nanomaterials the fluorescent dye can be covalently anchored either onto the silica surface or doped into the matrix of the silica shell^2,3,11–13^.

Electrospinning has been one of the most versatile methods employed for generating nano- and microfibers^14–16^. Its simplicity, scalability and high versatility renders this method very attractive in many scientific fields. Electrospun polymer-based organic-inorganic fibrous nanocomposites have been developed by many research groups and further evaluated in various fields including biomedicine^17–19^, catalysis^20–22^, sensing^23^, energy^24,25^ and environmental protection^26–29^. However, only a few examples appear to date on the fabrication of nanocomposite electrospun fibers with embedded core-shell ferrous nanoparticles^30,31^. In one such example, core-shell Fe/FeO nanoparticles have been incorporated within polyimide fibers aiming to produce fibrous nanocomposites exhibiting enhanced thermal stability and magnetic properties for potential use in high-temperature magnetic sensing and microwave absorption applications^30^.

The general advantages of combining superparamagnetic Fe_3_O_4_ nanoparticles with fibrous materials designed for use in sensing applications include among others the inherent ability of magnetic Fe_3_O_4_ nanoparticles to act as effective gas sensors^32^ and the possibility for employing fiber alignment *via* magnetic field-assisted electrospinning^33^. Concerning the latter, it has been demonstrated that fiber alignment results in the enhancement of the sensing properties compared to their randomly oriented analogues^34,35^. Furthermore, the magnetic properties of the produced electrospun mats could provide an additional functionality in their manipulation and processing. More precisely, it may promote the magnetic bonding of the fibrous mats on suitable electromagnetic holding platforms, the controllable remote heating of materials for specific sensing applications or the efficient collection of mats by magnetic means from remote areas in various sensing applications^36^. Additionally, magnetic electrospun mats with sensing capabilities could be employed as overlayers on various optical platforms in integrated optics or optical fibers. Such a thin magnetic overlayer on the proximity of an optical waveguide could operate as a sensing element of magnetic measurands that could alter the surrounding refractive index leading to pure photonic interrogation, with the simultaneous monitoring of pH conditions. Other applications may also include the measurement of magnetic fields by the induced deflection of fibers or waveguide cantilevers in the presence of magnetic fields, retaining at the same time the chemical sensing capability. Furthermore, in the presence of electric high frequency fields the resulted remote heating of overplayed mats could alter the refractive index properties of optical waveguides allowing thus the optical sensing of fields or temperature in magnetic hyperthermia applications^36^ and in cases where pH monitoring would also be necessary.

Herein, ferrous core-shell nanoparticles consisting of a magnetic γ-Fe_2_O_3_ multi-nanoparticle core and an outer SiO_2_ shell have been synthesized and further functionalized with Rhodamine B (RhB) fluorescent molecules (γ-Fe_2_O_3_/SiO_2_/RhB NPs). The latter were covalently bound in the matrix of the silica shell. The resulting γ-Fe_2_O_3_/SiO_2_/RhB NPs were further incorporated within cellulose acetate (CA) electrospun fibers to yield fluorescent multifunctional fibrous nanocomposites. Electrospun fibers with embedded fluorescence moieties designed for use in fluorescence sensing are considered to be highly advantageous compared to their film analogues due to their larger surface-to-volume ratios. In previous reports on fluorescent-functionalized electrospun polymer fibers, the fluorophores were either covalently attached onto the polymer backbone^37–41^ or incorporated as dopants within the fibers^42–46^.

In one such example referring to the doping of polymer nanofibers with RhB, the fluorescent dye was added into a poly(ether sulfone) solution prepared in N,N-dimethylacetamide and the mixture was electrospun to obtain fluorescent, RhB-doped nanofibers that were further evaluated as metal ion (Cu^2+^) fluorescent sensors in aqueous media^47^. Furthermore, electrospun polymer fibers doped with RhB derivatives have been successfully used as highly efficient turn-on fluorescent sensors for the detection of Hg^2+^ ions^48,49^.

In the present study, the use of γ-Fe_2_O_3_/SiO_2_/RhB NPs as dopants in electrospun fibers is considered to be advantageous compared to other fabrication routes reported so far, since the leakage of the RhB fluorophore from the core-shell NPs is prevented due to its covalent anchoring onto the nanoparticle surfaces. In contrast, small fluorescent molecules introduced within the polymer fibers as dopants are only held onto the polymer chains *via* weak van der Waals interactions, thus often resulting to their desorption from the polymer matrix and consequently the decrease in the fluorescence efficiency of the fibers. In addition, the covalent anchoring of RhB molecules onto the nanoparticles’ surfaces and the blending of the γ-Fe_2_O_3_/SiO_2_/RhB NPs with the fibrous CA matrix, suppress self‐quenching phenomena, whereas by covalently integrating RhB within the silica shell, fluorescence quenching is further prevented by avoiding direct contact with iron oxides.

Besides the above, the use of a renewable, naturally-abundant acetylated cellulose derivative as a polymer matrix exhibiting biocompatibility, biodegradability and environmental friendliness, combined with the magnetic character of the inorganic γ-Fe_2_O_3_/SiO_2_/RhB NPs additives providing the possibility for magnetic separation by applying an external magnetic field, are additional benefits of the multifunctional fibrous nanocomposite fluorescent sensor described in this study.

Two different fabrication protocols were followed for the preparation of CA fibers doped with the γ-Fe_2_O_3_/SiO_2_/RhB NPs. In the first synthetic route the nanoparticles were sprayed on top of the fibrous mat while in the second approach a nanoparticle suspension in the CA polymer solution was directly electrospun to yield the final product. The dual sensing capability (i.e. ammonia gas and pH sensing) of the developed fibrous nanocomposites was investigated for both types of the produced nanocomposites. In both cases, a good response for gas ammonia sensing was shown for high NH_3_ concentrations. The presented multifunctional electrospun ammonia sensors show high tolerance in poisoning and saturation effects, providing linear response up to 12000 ppm. Consequently, they could be extremely valuable in the detection of high levels of ammonia (concentrations above 1000 ppm) at industrial facilities employing ammonia transfer lines and dense storage spaces. Moreover, low cost sensing elements or materials can then be replaced after logging an ammonia leakage event.

In the case of pH sensing, the fibrous nanocomposites in which the NPs were embedded inside the fibers were more robust compared to the fibers having the NPs deposited *via* spraying onto their surfaces, with the former demonstrating a fast and linear response in both alkaline and acidic environments with good reversibility.

## Experimental

### Materials

For the synthesis of the γ-Fe_2_O_3_/SiO_2_/RhB NPs, reagent grade chemicals were used as received from the manufacturers. Iron (III) sulfate hydrate, iron (II) sulfate heptahydrate (ACS, 99%), citric acid (CA, 99%), tetraethoxysilane (TEOS, 99.9%) and NH_4_OH (28–30%) were supplied by Alfa Aesar (Lancashire, UK). Acetone (AppliChem GmbH) and absolute ethanol (Carlo Erba, reagent - USP) were used as received. (3**-**Aminopropyl)triethoxysilane (APS; silane-NH_2_, 99%), tetraethoxysilane (TEOS; 98%), ethyl acetate (EA), dichloromethane (DCM), dimethylformamide (DMF), Rhodamine B isothiocyanate (RhB), poly(acrylic acid) solution (25 wt. % in water) and polyvinyl pyrrolidone (PVP, 40 kDa) were obtained from Sigma-Aldrich (St. Louis, MO, USA).

Cellulose acetate (CA, Mn = 30000 g/mol) was obtained from Sigma-Aldrich and used without further purification. Acetone (Technical Grade 99.5% - Panaska Trading CO) was the solvent used in the preparation of CA polymer solutions that were further electrospun.

### Synthesis of γ-Fe_2_O_3_/SiO_2_ NPs

The core-shell γ-Fe_2_O_3_/SiO_2_ NPs were obtained by the self-assembly of primary superparamagnetic maghemite (γ-Fe_2_O_3_) nanoparticles (size ∼ 10 nm) followed by coating of the superparamagnetic nanoparticle clusters with a layer of silica^50–52^. In brief, maghemite nanoparticles were synthesized by co-precipitation of Fe^3+^/Fe^2+^ from an aqueous solution and transfer to ethyl acetate^53^. The nanoparticle clusters were prepared by emulsification of the ethyl acetate suspension (∼5 vol% of the continuous phase) as an inner phase with water as a continuous phase. The poly(acrylic acid) was mainly used as a capping agent/surfactant for nanoparticle stabilization. The residual ethyl acetate was removed using a rotary evaporator. Subsequently, the particles were washed with ethanol and distilled water. As a result, nanoparticle clusters containing ∼80–100 maghemite nanoparticles were formed^54,55^. All procedures were developed exclusively by the company Nanos SCI and they are described in detail in previous publications^50–55^.

### Synthesis of γ-Fe_2_O_3_/SiO_2_/RhB NPs

Rhodamine-B (RhB) was covalently integrated into the matrix of the silica shell as described below^4^.

The reaction between RhB and APS was carried out first in the mixture of DCM/DMF = 4/1 overnight at room temperature. RhB (0.00933 mmol) was dissolved in the solvent mixture (0.5 mL) and then APS (0.186 mmol) was added. Subsequently, the volatile solvent was removed using nitrogen flow and the product (RhB-APS) was mixed with TEOS for the silica coating. The general synthetic route is described as follows: First, 100 mg of PVP was dissolved in 200 mL of ethanol containing 450 mg of nanoparticle clusters. Subsequently, 1.2 mL of TEOS and RhB-APS was mixed and then added to the suspension followed by the addition of 2.0 mL of aqueous ammonia solution. The reaction mixture was stirred using a 2-cm-wide glass propeller at 300 rpm for six hours at room temperature and upon completion, the product was washed first with ethanol and then 3-times with distilled water. Finally, an additional thin layer (a few nanometers thick) of non-fluorescent silica was deposited using the same synthetic protocol with the only difference being the amount of TEOS, i.e. 0.15 mL of TEOS in the absence of RhB-APS. The prepared γ-Fe_2_O_3_/SiO_2_/RhB NPs were dispersed in ethanol-based stable colloidal suspensions at a concentration of 26 mg/mL.

### Fabrication of γ-Fe_2_O_3_/SiO_2_/RhB NPs-functionalized electrospun CA fibers

Initially, CA (2.5 g) was dissolved in acetone (20 mL) upon stirring at ambient conditions and the obtained colourless transparent homogeneous solution was loaded in the syringe of the electrospinning set-up. All electrospinning experiments were performed at room temperature. Equipment included a controlled-flow, four-channel volumetric microdialysis pump (KD Scientific, Model: 789252), a syringe with a connected spinneret needle electrode, a high-voltage power source (10–50 kV) and a custom-designed grounded target collector inside an interlocked Faraday enclosure safety cabinet. Systematic parametric studies were carried out by varying the applied voltage, the needle-to-collector distance, the needle diameter and the flow rate so as to determine the optimum experimental conditions for obtaining CA nanoparticle-free fibers. The electrospinning conditions used for obtaining continuous, nanoparticle-free fibers were the following: Flow rate: 5.9 mL/hr; applied voltage: 15 kV; needle-to-collector distance: 10 cm; Needle diameter: 16G.

Fabrication of CA/γ-Fe_2_O_3_/SiO_2_/RhB NPs nanocomposite fibers was carried out by following 2 different experimental protocols. In the first one, a CA fibrous mat (19.5 mg) was placed inside a petri dish and the nanoparticle suspension (1 mL, nanoparticle suspension in ethanol, concentration: 26 mg/mL) was sprayed on top of the fibrous mat. Afterwards, the fibers were placed in a laboratory fume hood until complete drying. The 2^nd^ experimental protocol involved the dropwise addition of 1 mL of the aforementioned nanoparticle suspension in the CA polymer solution prepared in acetone (polymer solution concentration: 12.5% w/v) during stirring at ambient temperature and subsequent electrospinning of the resulting suspension under identical electrospinning conditions applied in the case of the CA fibers, to obtain CA/γ-Fe_2_O_3_/SiO_2_/RhB NPs nanocomposite fibers.

### Materials characterization

For the TEM investigations, the γ-Fe_2_O_3_/SiO_2_/RhB NPs were deposited by drying dispersion on a copper-grid supported, perforated, transparent carbon foil and analysed by TEM (Jeol, JEM, 2100) which operated at 200 kV. The magnetic properties of the samples were measured at room temperature by Vibrational Sample Magnetometry (VSM) (Lake Shore 7307 VSM). The dry, γ-Fe_2_O_3_/SiO_2_/RhB NPs-decorated CA fibers (10–15 mg) were placed into the VSM system prior to measurements. Magnetic characterization was carried out at room temperature. The hydrodynamic size distributions of the core-shell nanoparticles were measured using dynamic light scattering (DLS, Fritsch, Analysette 12 DynaSyzer, Germany).

The morphological characteristics of the produced fibers prepared in the absence and presence of the γ-Fe_2_O_3_/SiO_2_/RhB NPs were determined by scanning electron microscopy (SEM) (Vega TS5136LS Tescan). The samples were gold-sputtered (sputtering system K575X Turbo Sputter Coater – Emitech) prior to SEM inspection.

Fluorescence microscopy was further used for visualizing the pristine and γ-Fe_2_O_3_/SiO_2_/RhB NPs-modified CA electrospun fibers. The samples were placed on glass slides, covered with coverslips and documented under the Olympus fluorescence microscope (U-RLF-T model). The fluorescence intensity of the pure CA fibers was determined by using the FITC filter (Excitation: 490 nm, Emission: 520 nm) whereas for the γ-Fe_2_O_3_/SiO_2_/RhB NPs-modified fibers a CY3 filter was used (Excitation: 552 nm, Emission: 570 nm). Images were analyzed using the cellSens software.

### Ammonia and pH sensing apparatus

**Figure 1:**
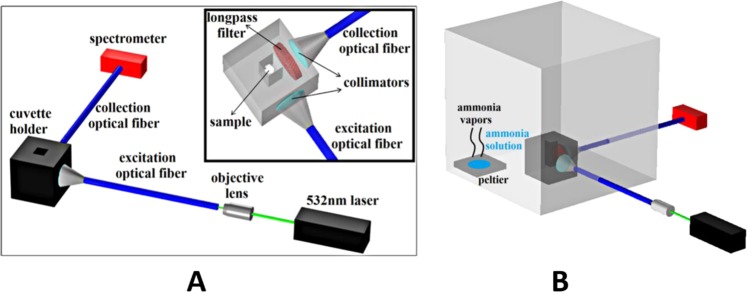
Schematic presentation of the experimental setup employed in ammonia gas sensing and pH sensing experiments (**A**) and of the ammonia gas sensing set-up (**B**).

In order to measure the samples’ response to ammonia vapors and different pH values, the γ-Fe_2_O_3_/SiO_2_/RhB NPs-functionalized CA electrospun fibers were placed in a cuvette holder having two perpendicular light paths that were specially designed for free space applications. An all solid state 532 nm laser was used to excite the RhB moiety using a 400 μm core multimode silica optical fiber. The emitted fluorescence was filtered by a Thorlabs FGL550 longpass filter with 550 nm cutoff wavelength that blocks the excitation wavelength. It was then collected by a 600 μm core multimode optical fiber and analyzed by a Thorlabs CCS200 spectrometer. Both SMA terminated optical fibers are connected with the cuvette with SMA fiber adapters having mounted collimators. A schematic representation of the setup is shown in Fig. 1A.

For the gas ammonia detection, the above described cuvette holder containing the sample was placed in a custom made sealed testing chamber of 4.3 L volume. A peltier element was used for the evaporation of the 25% w/v ammonia solution drops as schematically depicted in Fig. 1B. For the pH detection, aquatic solutions of different pH values were used. HCl solutions with pH values ranging from 1–5 and NaOH solutions ranging from 8–13 were inserted in the cuvette where the sample was placed, in order to evaluate the sample’s response.

## Results and Discussion

### Synthesis and characterization of γ-Fe_2_O_3_/SiO_2_/RhB NPs

**Figure 2:**
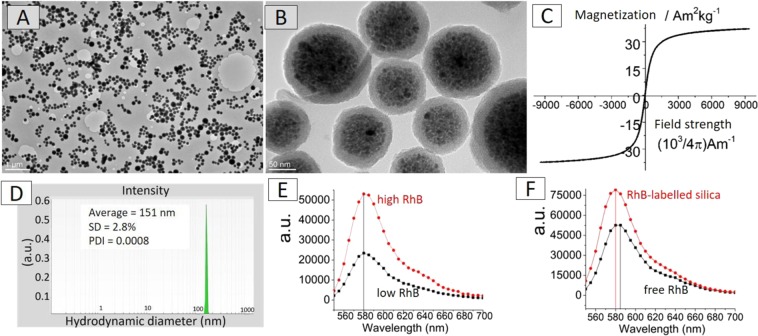
TEM images of γ-Fe_2_O_3_/SiO_2_/RhB NPs at low (**A**) and high (**B**) magnification, room-temperature measurement of the magnetization as a function of magnetic field strength (**C**), and intensity-weighted distribution of the γ-Fe_2_O_3_/SiO_2_/RhB NPs hydrodynamic diameters obtained from the DLS measurements in ethanol-based suspension at concentration 1 mgmL^−1^ (**D**). Emission spectra of γ-Fe_2_O_3_/SiO_2_/RhB NPs with high (9.33 µmol of RhB) and low (3.11 µmol of RhB) RhB loading (**E**). Emission spectra of the γ-Fe_2_O_3_/SiO_2_/RhB NPs and of the aqueous solution containing free RhB molecules (**F**).

Fluorescent silica-coated nanocrystal clusters with approx. 20 nm thick silica shell were prepared as fluorescent and magnetic labels for the produced CA electrospun fibers (γ-Fe_2_O_3_/SiO_2_/RhB NPs). The magnetic cores of the γ-Fe_2_O_3_/SiO_2_/RhB NPs were prepared by the self-assembly of approximately a hundred of superparamagnetic maghemite nanocrystals (size ∼10 nm). Darker magnetic nanocrystal cores can be clearly distinguished from the brighter amorphous silica shell in the TEM images (Fig. 2A,B). The size of the γ-Fe_2_O_3_/SiO_2_/RhB NPs determined from the TEM images (>100 particles counted) was found to be ∼130 nm ± 30 nm. The γ-Fe_2_O_3_/SiO_2_/RhB NPs showed superparamagnetic properties with a saturation magnetization Ms of ∼37 Am^2^ kg^−1^ (Fig. 2C). DLS measurements of the γ-Fe_2_O_3_/SiO_2_/RhB NPs in an ethanol suspension (1.0 mg mL^−1^) showed narrow hydrodynamic-size distribution with the average size at ∼151 nm (SD = 2.8%) (Fig. 2D).

The γ-Fe_2_O_3_/SiO_2_/RhB NPs form stable colloidal suspensions and this is verified by the fact that their DLS-determined size is in close agreement with the size determined by TEM.

In order to investigate the influence of the RhB-loading on the nanoparticles’ fluorescence properties, γ-Fe_2_O_3_/SiO_2_/RhB NPs having a lower RhB amount (i.e. only 3.11 µmol of RhB instead of 9.33 µmol per 450 mg of γ-Fe_2_O_3_/SiO_2_/RhB NPs – see in section 2.3) were synthesized. As seen in Fig. 2E, the emission spectra of the γ-Fe_2_O_3_/SiO_2_/RhB NPs with high RhB loading showed higher relative intensity compared to the low RhB loading clusters as expected. Most importantly, no shift in the maximum emission wavelength was observed upon altering the RhB loading.

In order to examine whether the optical response of RhB is influenced by the covalent linkage to the silica shell, the maximum emission wavelengths of the γ-Fe_2_O_3_/SiO_2_/RhB NPs and of the RhB aqueous solution (free RhB) were compared. As seen in Fig. 2F, the maximum emission wavelength of RhB aqueous solution was recorded at 585 nm, while after covalent anchoring with silica, the wavelength was shifted to 580 nm. It is noteworthy to mention at this point that when RhB was simply added to the TEOS (silica precursor) without the pre-formation of a covalent bond with the amino-silane molecule (APS), the product (silica-coated nanoparticle clusters) was not fluorescently labelled and all RhB molecules were removed during vigorous washing.

### Fabrication of γ-Fe_2_O_3_/SiO_2_/RhB NPs-functionalized electrospun CA fibers

**Figure 3:**
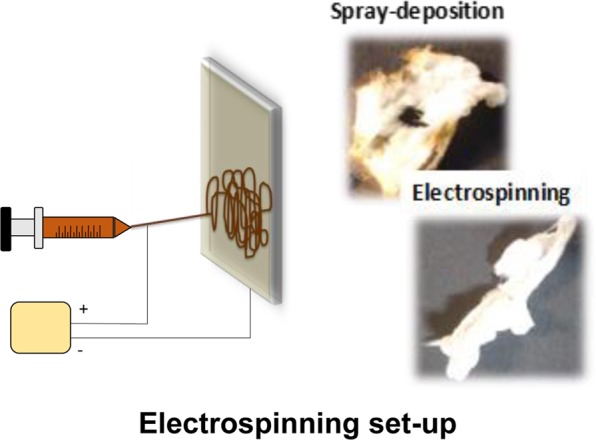
Left: Schematic of the electrospinning set-up used in the fabrication of electrospun CA fibers. Right: Indicative photographs of the γ-Fe_2_O_3_/SiO_2_/RhB NPs-functionalized CA fibers obtained *via* spray deposition of the γ-Fe_2_O_3_/SiO_2_/RhB NPs onto the fibers’ surfaces – sprayed magnetic fibers – (up) and by mixing of the γ-Fe_2_O_3_/SiO_2_/RhB NPs dispersion with the polymer solution prepared in acetone, followed by electrospinning – electrospun magnetic fibers – (down).

Electrospinning was first employed in the fabrication of pure CA fibers. A schematic of the electrospinning set-up used is provided in Fig. 3. The experimental conditions employed for obtaining nanoparticle-free CA electrospun fibers are given in the experimental section.

**Figure 4:**
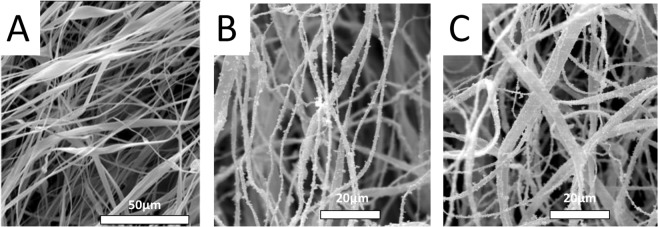
SEM images of the as prepared CA fibers (**A**), the sprayed magnetic fibers (**B**) and the electrospun magnetic fibers (**C**).

The morphological characteristics of the produced CA fibers were investigated by SEM. As seen in the SEM images appearing in Fig. 4. CA fibers had a belt-like (ribbon-like) morphology, a random orientation and they were characterized by a relatively broad diameter distribution within the micrometer size range. Ribbon-like morphologies in electrospun fibers based on cellulose and cellulose derivatives have been previously reported^56^. Based on earlier reports, the CA fiber morphology can be altered upon changing the solvent system and the polymer solution concentration. Under certain experimental conditions (i.e. specific solvent system, polymer solution concentration and optimum electrospinning parameters) the generation of cylindrical CA fibers is also feasible^57^.

As already mentioned in the experimental section, the fabrication of CA electrospun fibers decorated with γ-Fe_2_O_3_/SiO_2_/RhB NPs was accomplished by following two different synthetic routes involving: (a) the deposition of the nanoparticles onto the fibers’ surfaces *via* spraying (denoted as sprayed magnetic fibers) and (b) the mixing of the nanoparticle dispersion prepared in ethanol with the CA acetone solution followed by electrospinning (denoted as electrospun magnetic fibers).

RhB was chosen to be covalently linked onto the nanoparticles’ surfaces due to its high photostability compared to other fluorescent dyes introduced in previous studies as active moieties in optical sensing applications^58^. Actually, earlier studies in our group involved the incorporation of fluorescein (FL)-functionalized core-shell ferrous nanoparticles (γ-Fe_2_O_3_/SiO_2_/FL NPs) within CA electrospun fibers by following the spraying deposition methodology which however demonstrated low photostability, in agreement with previous reports^59^.

The γ-Fe_2_O_3_/SiO_2_/RhB NPs-functionalized CA fibers were visualized by SEM and fluorescence microscopy. As seen in Fig. 4(B,C), in both cases, the presence of the nanoparticles on the fibers’ surfaces can be clearly seen.

**Figure 5:**
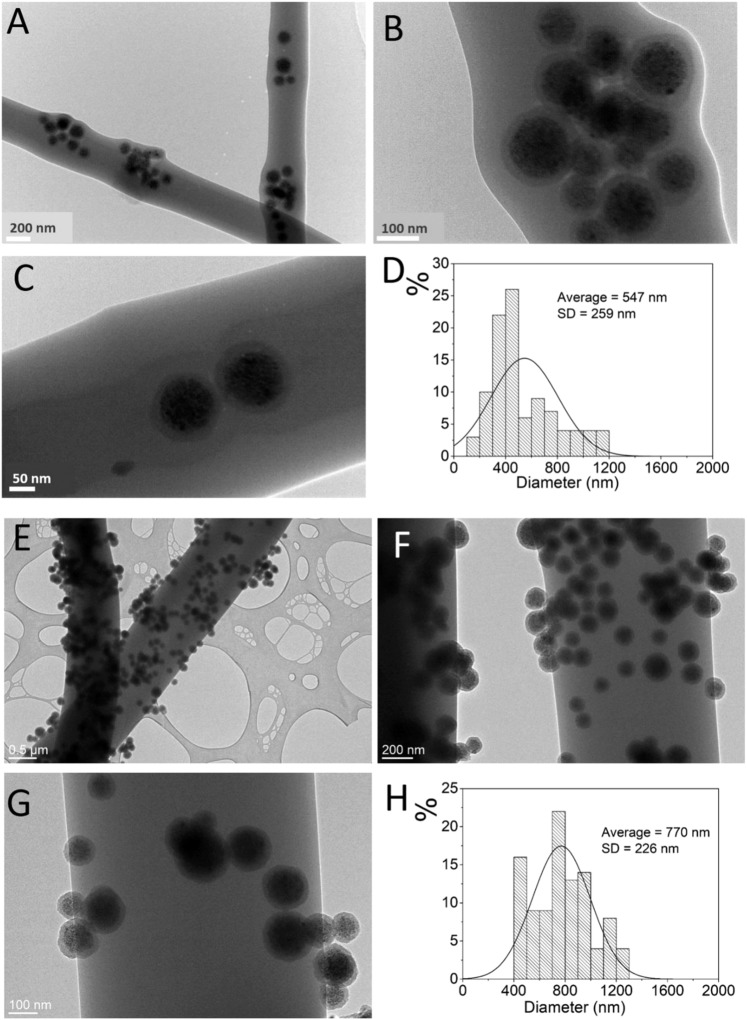
TEM images of the electrospun magnetic fibers (**A**–**C**) and the sprayed magnetic fibers (**E**–**G**). Fibers diameter distributions were determined by analysis of the TEM images corresponding to the electrospun magnetic fibers (**D**) and the sprayed magnetic fibers (**H**).

Transmission electron microscopy (TEM) analyses confirmed the presence of γ-Fe_2_O_3_/SiO_2_/RhB NPs along the nanocomposite fibers (Fig. 5). By comparing the TEM images of the electrospun magnetic fibers (Fig. 5A–C) with the sprayed magnetic fibers (Fig. 5E–G), the presence of the Fe_2_O_3_/SiO_2_/RhB NPs exclusively onto the fibers’ surfaces can be observed in the second case, in contrary to the electrospun magnetic fibers where the nanoparticles are mainly accumulated within the fibers. Moreover, the synthetic protocol affected the mean fibers’ diameter significantly where electrospun magnetic fibers’ approach yields thinner while sprayed magnetic fibers’ approach yields thicker fibers with diameter of 547 nm and 770 nm, respectively.

In the case of the electrospun materials, the core-shell nanoparticle morphology could clearly be resolved in images at higher magnifications (Fig. 5B,C). The Fe_2_O_3_/SiO_2_/RhB NPs are relatively homogeneously distributed along the fibers while on the nanoscale there is some segregation composed of up to dozen nanoparticles present in small aggregates.

**Figure 6:**
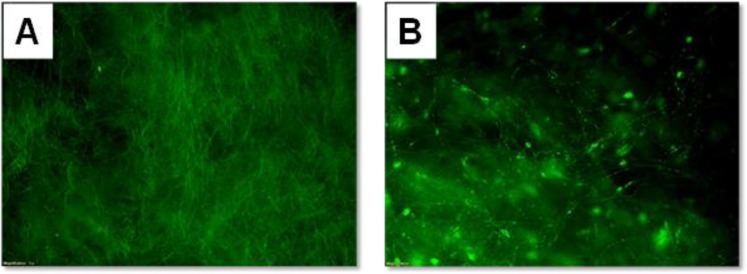
Fluorescence microscopy image of the sprayed magnetic fibers (**A**) and the electrospun magnetic fibers (**B**).

Fluorescence microscopy was used to verify the fluorescence properties of the multifunctional nanocomposite fibers. Figure 6 provides the fluorescence images of the produced materials. By observing the fluorescence microscopy images it can be seen that fluorescence is not homogeneous for the entire sample, particularly in the case of the electrospun magnetic fibers (Fig. 6B). According to the TEM data (provided in Fig. 5), the sprayed magnetic fibers have Fe_2_O_3_/SiO_2_/RhB NPs anchored all over their external surfaces. In contrary, in the case of the electrospun magnetic fiber analogues, the nanoparticles that are embedded within the CA fibers form clusters and the presence of nanoparticle-free regions along the fibers can be clearly observed, resulting to a fluorescence “inhomogeneity” along the fibers.

**Figure 7:**
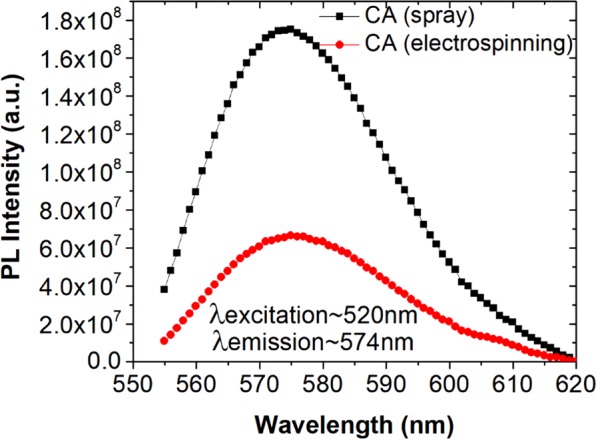
Photoluminescence spectra of the γ-Fe_2_O_3_/SiO_2_/RhB NPs-functionalized CA fibers obtained *via* spray deposition (via spray deposition and electrospinning) (excitation wavelength: 520 nm).

The fluorescence efficiency of the Fe_2_O_3_/SiO_2_/RhB NPs anchored onto the CA fiber surfaces was investigated by photoluminescence spectroscopy at 520 nm excitation wavelength (Fig. 7). The emission wavelength was recorded at 574 nm, in agreement with previous reports recording the emission wavelength of RhB within 574–577 nm^60–62^.

**Figure 8:**
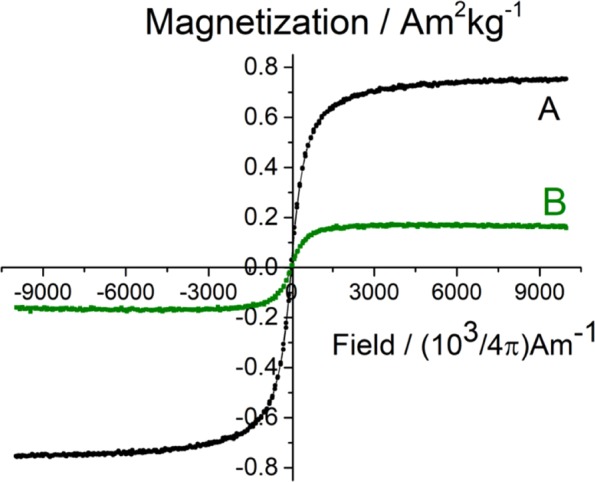
A room-temperature measurement of the magnetization as a function of magnetic field strength of the γ-Fe_2_O_3_/SiO_2_/RhB NPs-functionalized CA fibers obtained *via* spray deposition (**A**) and electrospinning (**B**).

The magnetic behaviour of the nanocomposite membranes was investigated by VSM at room temperature. Figure 8 presents the magnetization *versus* applied magnetic field strength plots for the 2 types of the γ-Fe_2_O_3_/SiO_2_/RhB NPs-functionalized CA nanocomposite fibers. As seen in the plots, both systems exhibited superparamagnetic behavior at ambient temperature, demonstrated by the symmetrical sigmoidal shape of the magnetization curves and the absence of a hysteresis loop. The sprayed magnetic fibers had a higher saturation magnetization value (Ms ∼ 0.76 Am^2^ kg^−1^) compared to the electrospun magnetic fibers (Ms ∼ 0.20 Am^2^ kg^−1^) due to the presence of the non-magnetic coating (cellulose acetate) around the NPs embedded within the fibers (in contrast to the NPs that are deposited onto the fiber surfaces), resulting to the decrease in magnetization due to quenching of surface effect^63^. Moreover, based on VSM magnetic measurements, the amounts of the γ-Fe_2_O_3_/SiO_2_/RhB NPs in the functionalized CA nanocomposite fibers were estimated to be ∼2.1 wt. % and ∼0.5 wt. % for the sprayed magnetic fibers and the electrospun magnetic fibers respectively. These differences justify the differences observed in the photoluminescence spectra corresponding to the 2 cases (Fig. 7).

### Gas (ammonia) and pH sensing

Both electrospun and sprayed magnetic fibers were evaluated for different gas ammonia concentrations and pH values. The emission spectrum of the sample was stable upon continuous illumination, showing no self-quenching mechanisms observed with other fluorescent moieties such as anthracene^41^. For the measurements, the integration time of the spectrometer was set to 10 s. The averaging method, rolling average over the last 3 acquisitions, was used. The sample was exposed for 30 s for each data point, showing fast response to the measurands. As each individual measurement corresponds to 30 s, this can be an estimate of the time response which is comparable to other electrospun-based ammonia sensors^64,65^ where the response times range between 50 s–350 s, as well as fluorescence-based sensors^66^. In that sense, the response of the materials presented herein can be considered as fast. In order to assure the reliability of measurements for each recorded value, the sample was illuminated for 10 more minutes before changing NH_3_ concentration or pH, showing no further change. This stability demonstrates that at continuous excitation, the properties of the material at constant ammonia concentration were not deteriorated and also that the response is attributed reliably to the specific measuring conditions.

**Figure 9:**
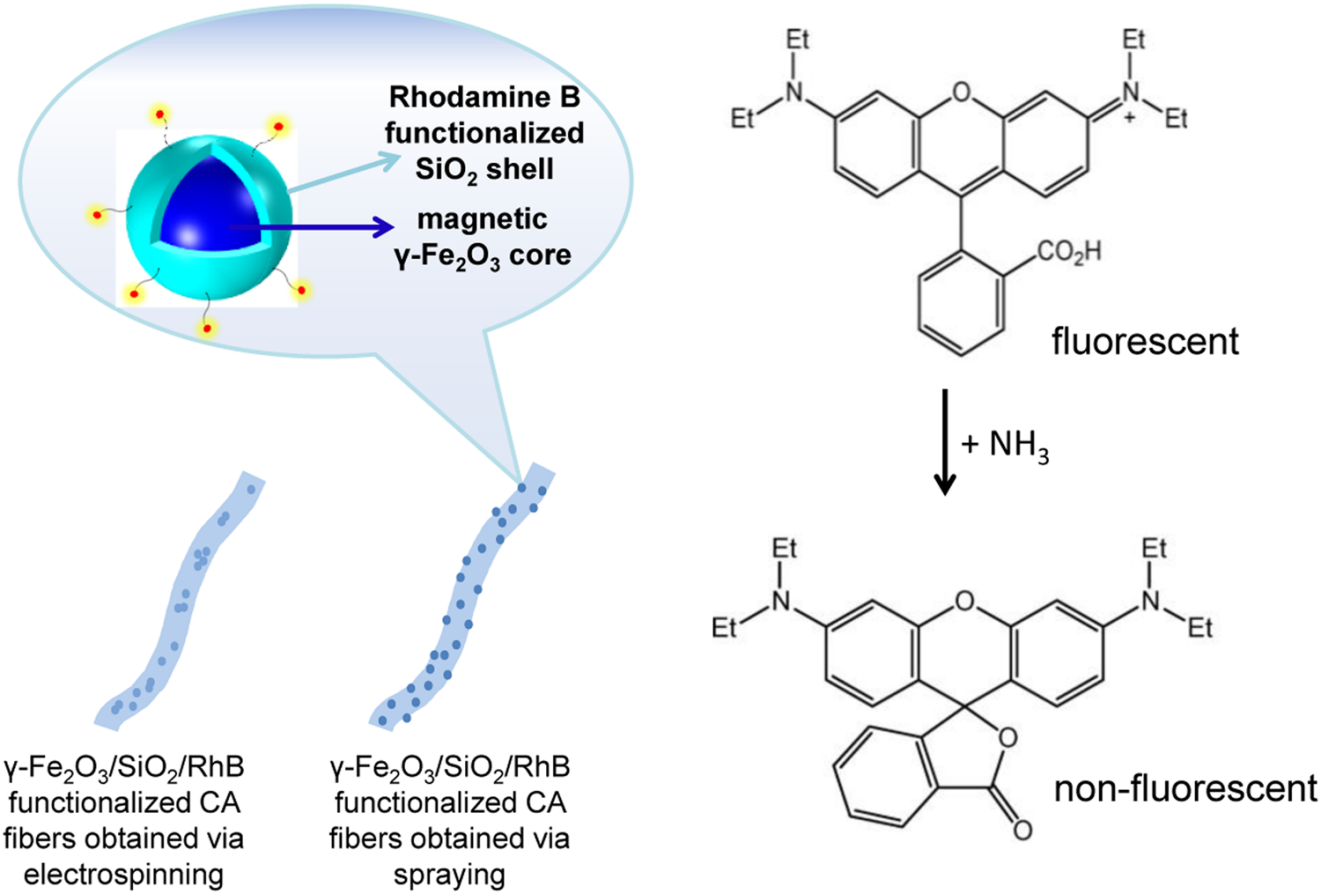
Sensing (fluorescence quenching) mechanism of RhB molecules undergoing structural changes when exposed to NH_3_ vapours, resulting to the generation of the non-fluorescent lactone form.

**Figure 10:**
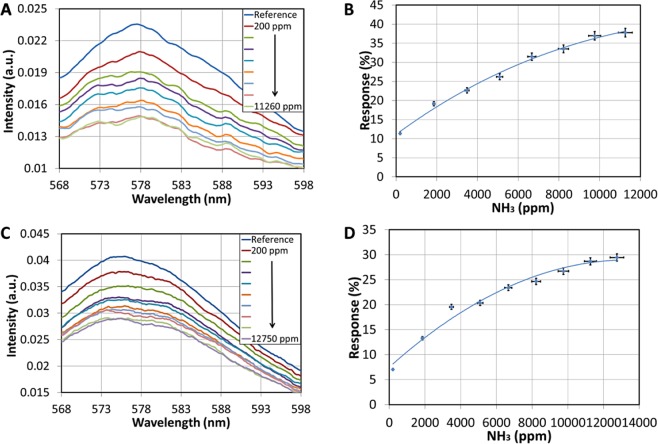
(**A**) Fluorescence spectra of the electrospun magnetic fibers for different concentrations of ammonia gas. (**B**) Response of the electrospun magnetic fibers at 577 nm for different ammonia concentrations. (**C**) Fluorescence spectra of the sprayed magnetic fibers for different concentrations of ammonia gas. (**D**) Response of the sprayed magnetic fibers at 577 nm for different ammonia concentrations.

When exposed to NH_3_ vapors, RhB undergoes structural changes resulting to the generation of a non-fluorescent lactone (Fig. 9)^67^. The latter explains the reduction observed in the fluorescence intensity upon exposure of the nanocomposite γ-Fe_2_O_3_/SiO_2_/RhB NPs -functionalized fibrous mats in NH_3_ (Fig. 10A,C).

Figure 10A shows the fluorescence spectrum of the electrospun magnetic fibers taken for different ammonia concentrations. A reference intensity value (*I*_*ref*_) was taken before inserting the ammonia vapors. The fibers’ response is defined as:

1 $$Response\,( \% )=\frac{{I}_{sig}-{I}_{ref}}{{I}_{ref}}\times 100$$

where *I*_*sig*_ is the intensity measured for an ammonia concentration. Figure 10B shows the response corresponding to the 577 nm peak, demonstrating a clear ammonia sensing for concentrations up to 11000 ppm.

The sprayed magnetic fibers decorated on their external surfaces with γ-Fe_2_O_3_/SiO_2_/RhB NPs also show a clear response to the ammonia vapours as presented in Fig. 10C,D for NH_3_ concentrations up to ~13000 ppm. The detection of high NH_3_ concentrations can be attributed to the large surface-to-volume ratio of the electrospun fibers. Previous studies have shown that due to their large surface-to-volume ratio, electrospun fiber sensors exhibit enhanced sensitivity^67^ as well as quenching efficiency^38^ compared to thin films. The overall factor that enables the high concentration detection is actually the total number of sensing elements which are the γ-Fe_2_O_3_/SiO_2_/RhB NPs. Their total number in a specific volume of the material is determined by the three dimensional (3D) fibrous form and the concentration of the functionalised nanoparticles. However, the surface enhancement enabled by the fibrous morphology is the dominant factor which can be considered as a nonlinear scaling factor in a three dimensional space where the nanofibrous material is organised. The arrangement of nanoparticles in a linear shaped fiber scales linearly but in this 3D fibrous form the total effect can be eventually characterised by a nonlinear scaling or enhancement factor.

The response of the sprayed magnetic fibers is ~5% lower compared to the electrospun magnetic fibers. This could be attributed partially to the fact that in the former case the NPs can easily detach from the fibers’ surfaces before and during the measurements because they are only weakly attached to them.

The sample starts to quench above ~12000 ppm and the response curve reaches a plateau. Therefore the sensor can be used for NH_3_ concentrations detection up to ~12000 ppm. Above this limit, the sensor cannot be reliably used as it becomes permanently “poisoned” and irreversible.

Furthermore, our experimental data show no consistent reversibility in high concentrations ammonia sensing. This can be attributed to the high ammonia concentration, as heavy loading (with ammonia) tends to destabilize the sensor, resulting in poor reversibility and smaller relative signal changes^68^. However, despite the non-reversible nature of the demonstrated behaviour, there are several sensing and detection applications where reversibility is not required as the purpose of the sensing element is to log/register successfully a specific high concentration of ammonia. Such cases are important in ammonia leakage monitoring especially at very high concentrations that can become toxic. At the current stage of development the applications of detection of high level of ammonia concentration (above 1000 ppm) could be used at industrial facilities employing ammonia transfer lines and dense storage spaces, as the presented sensors can withstand the poisoning and saturation effects and can thus provide linear response up to 12000 ppm. Low cost sensing elements or materials can then be replaced after logging an ammonia leakage event.

Although the physical form of the electrospun fibrous materials is suitable for high concentration gas detection, it is not considered ideal for efficient collection of fluorescent light that could enable low level of detection. This is attributed to the fact that the material arrangement in the measuring cell could be vulnerable to external factors like vibrations, air flow etc., resulting to possible displacements that may alter significantly the optimal excitation and collection light angles. Therefore, for the optimization of the sensing performance the development of a more robust customised miniaturised measuring apparatus is required in future studies. Furthermore, modification of the electrospun fibrous materials towards the development of electrospun rigid fibrous mats will facilitate in future studies a highly controllable characterization process in lower ammonia concentrations by improving also the optical excitation and collection system.

The γ-Fe_2_O_3_/SiO_2_/RhB NPs-functionalized CA fibers were also evaluated as pH sensors in aqueous solutions with different pH values. Both, electrospun and sprayed magnetic fibers were tested but only the former exhibited a consistent response/behaviour in aquatic environments. On the contrary, detachment of the γ-Fe_2_O_3_/SiO_2_/RhB NPs decorating only the surface of the sprayed magnetic fibers was observed when the latter were immersed in aquatic solutions, thus indicating the limited robustness of this system compared to the electrospun analogue. These results may be attributed to the weak nanoparticle/polymer matrix interactions arising from the surface functionalization *via* spray deposition. The latter is further supported by the TEM analysis provided in Fig. 5E–G.

**Figure 11:**
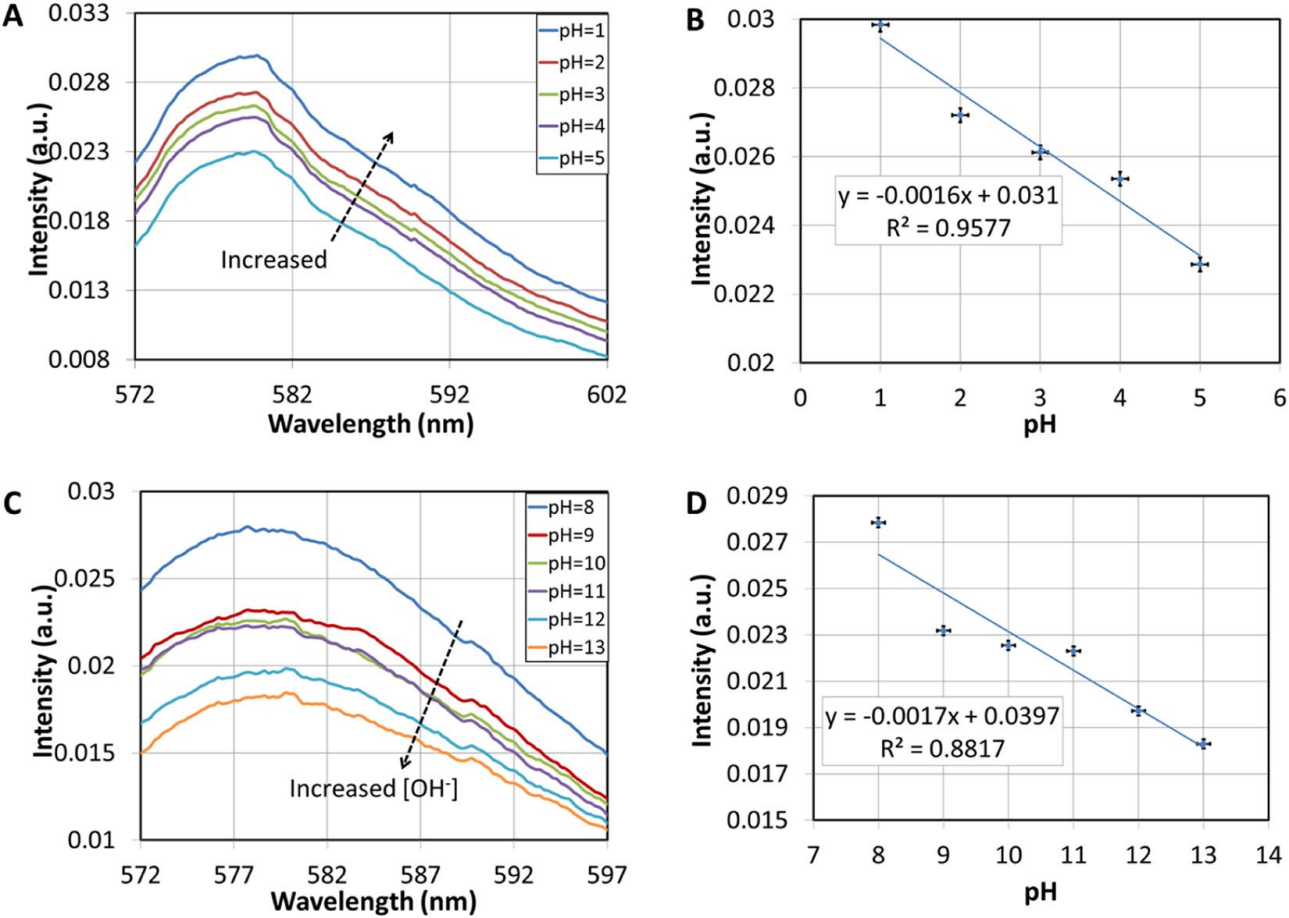
(**A**) Fluorescence spectra of the electrospun magnetic fibers for acidic aqueous solutions with different pH values. (**B**) Fluorescence intensity at 580 nm versus pH. **(C**) Fluorescence spectra of the electrospun magnetic fibers for basic aqueous solutions with different pH values. (**D**) Fluorescence intensity at 580 nm versus pH.

In Fig. 11A, the fluorescent intensity of the electrospun magnetic fibers immersed in acidic aqueous solutions with different pH values, ranging from 5 to 1, is presented. The fibers were firstly immersed in a pH 5 solution and subsequently in solutions with lower pH values. As the pH decreases, the fluorescent intensity clearly increases.

The electrospun magnetic fibers were also tested as fluorescent sensors in alkaline aqueous solutions with different pH values ranging from 8 to 13. The fibers were firstly immersed in a pH 8 solution and subsequently in solutions with higher pH values. As the pH increases the fluorescent intensity decreases (Fig. 11C,D). The intensity at the 580 nm peak is linearly depended on the pH value with R-square values of 0.9577 and 0.8817 for the acid and alkaline environments, respectively (Fig. 11B,D). The values of the slopes for the acid and alkaline environments are −0.00158 and −0.00165, respectively, with corresponding standard deviations of 0.0001917 and 0.0003028. The fact that the standard errors are less than 20% of the slope values strongly indicates that the correlation is linear. It should be noted that as the characterization of response in alkaline and acidic environments was performed by two different set of experiments due to current experimental limitations, the measurements cannot be directly compared or related in terms of the measured intensity, because of different placement conditions of the electrospun material in the measuring apparatus. However, the behaviour of the material is consistent over the entire pH range.

**Figure 12:**
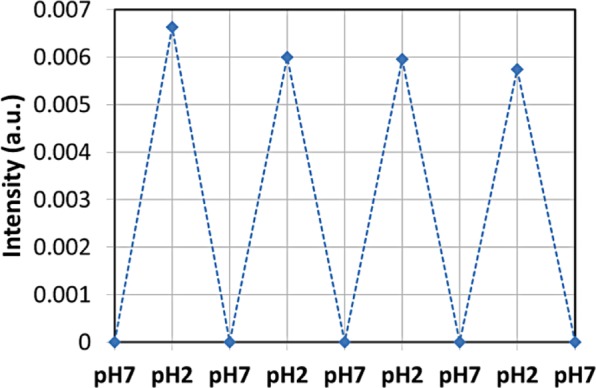
Reversibility of the pH-dependent on-off-on fluorescence intensity profile of electrospun magnetic fibers. The intensity was normalised to the intensity measured at pH = 7. The response time of the pH sensor is 30 s as estimated by the fluorescence measurements instrumentation.

The samples were also evaluated towards pH sensing reversibility by alternating solutions with pH values of 2 and 7. The sample exhibited reversible on/off switchable fluorescence emission as presented in Fig. 12 in accordance to the literature^69,70^. It is noteworthy to emphasize that the γ-Fe_2_O_3_/SiO_2_/RhΒ NPs–functionalized electrospun magnetic fibers characterised 6 months after production towards pH sensing, exhibited a measurable response for a relatively long range of pH between 2 and 7, together with a consistent reversibility, demonstrating their stability and long term functionality. The relatively small degradation of response signal could be attributed to either a drift or poisoning effect, or also to combined random changes in the environmental conditions and setup’s parameters during the long duration (~120 min) of the experiment. Despite this degradation the discrimination between the two pH values is still very clear and reliable.

To summarize, concerning the ammonia sensing performance, the sprayed magnetic fibers showed a ~5% lower response compared to the electrospun analogues, partially due to nanoparticle concentration differences in the two samples deriving from the fact that in the case of spray deposition the NPs are detached from the fibers’ surfaces before and during the measurements because they are only weakly attached to them.

Further to the above logical assumption, it should be underlined that the absolute value of NPs concentration and the resulted photoluminescence (Fig. 7) of the two different fiber types should not be related to their sensing responses, as the response is calculated to a reference intensity value (Eq. 1) connected to the absolute initial photoluminescence. The absolute photoluminescence is reflected only by the absolute values of the measured intensity in the graphs appearing in Fig. 10A,C, which are indeed higher for the case of the sprayed magnetic fibers (Fig. 10C). Therefore, it is noteworthy to stress out that the response in the case of the electrospun magnetic fibers (Fig. 10B) is higher despite the fact that the sensing NPs are embedded in the hosting fibers and are not directly exposed to ammonia gas as in the case of the NPs deposited onto the fibers’ surface *via* spraying. The adsorption mechanism of ammonia in the electrospun magnetic fibers is proved equivalent (or even more efficient), compared to the direct sensing on the sprayed NPs because of the minimal dimension of fibers. Furthermore, the stability of the NPs is retained, since these are protected within the fibers and they are not directly exposed to external degradation factors.

Based on the above, the sprayed magnetic fibers were found to be less robust as pH fluorescent sensors owned to the fact that the γ-Fe_2_O_3_/SiO_2_/RhB NPs decorating only the fibers’ surfaces were detached upon immersion of the fibers in aquatic solutions indicating their ineffectiveness in applications involving aquatic environments.

## Conclusions

In this work, the fabrication and characterization of cellulose acetate electrospun fibers doped with γ-Fe_2_O_3_/SiO_2_/RhB NPs is reported. Two different fabrication protocols were followed. In the first synthetic approach, the γ-Fe_2_O_3_/SiO_2_/RhB NPs were sprayed on top of the fibrous mat while in the second, a NP suspension prepared in CA acetone solution was directly electrospun. In the latter case, due to the encapsulation of the NPs within the fibers and the covalent anchoring of the RhB fluorophore onto the NP surfaces, the fluorophore’s leakage from the fibrous mat is prevented, enabling thus stable fluorescence-based operation of the developed materials.

In ammonia sensing performance, the sprayed magnetic fibers show a response ~5% lower compared to the electrospun magnetic fibers. It should be stressed out that the electrospun magnetic fibers exhibit equivalent or even higher response despite the fact that the sensing γ-Fe_2_O_3_/SiO_2_/RhB NPs are embedded in the hosting fibers and not directly exposed to ammonia gas as in the case of the sprayed analogues, while - as mentioned above - the embedded NPs retain also a much better stability since they are protected within the fibrous polymer matrix.

The electrospun nanocomposite fibers were evaluated for both gas ammonia and pH sensing. Due to the large surface to volume ratio of the functionalized fibrous mats, high ammonia concentrations up to 12000 ppm were detected. Furthermore, the electrospun magnetic fibers showed fast and linear response to aquatic solutions of long pH range from acidic to alkaline environments. However, the stability of the sprayed magnetic fibers is limited due to the detachment of the Fe_2_O_3_/SiO_2_/RhB NPs from their surfaces.
